# Tomatoes

**Published:** 1881-03

**Authors:** 


### TOMATOES.

This excellent and healthful vegetable ought to be eaten freely in every family. All who can do so should grow enough for their own use, as it is important that tomatoes be eaten in as fresh a condition as possible. They may be had early, even without a hot-bed, by sowing seeds in a box in the house at any time in March. The box should be kept in an east or south window during the day. At night it is better to place it in the centre of the room in order to secure greater warmth. Water moderately with warm water every day until the seeds begin to sprout. When the plants are about two inches high, select a few of the strongest and transplant to small pots, one plant in the middle of each pot, and keep from the light for a day or two. There is nothing objectionable in having a few pots of tomato plants, among the geraniums and roses of the bay window. In fact, it is an advantage "all around," as the tomato, in such a position, is not so apt to be neglected, and its strong odor has a tendency to keep insects away from the delicate plants that are more pleasing to us. Transplanting to the open ground may be done about the middle of May, or as soon as warm weather sets in. All of the plants should not be set at once, as some may fail, and it will be convenient to fill their places from those reserved in the house. The plants should be protected from the sun for a few days, or until strong enough to bear it. For this purpose, drain-tile, or pieces of stove-pipe may be used, but must be removed at night, if there be no danger from chilling winds. If the light is excluded too long, the plants will become weak. Tomato vines do well if trained on the south side of a board fence. If planted in the garden they should be supported by trellises. Stakes driven firmly into the ground will do, if the vines are tied to them with wide strips of soft cloth.

One who has had no experience in tomato culture hardly knows which kind to choose, from the many varieties offered by seedmen. After experimenting with several "novelties," the writer is satisfied that nothing need be better than the old-fashioned "early smooth red." Uneven, wrinkled tomatoes are not favorites with the housekeeper, who has to prepare them for the table.

The following recipe for "tomato soup" is always timely, as canned tomatoes can be used in place of those growing in season.

TOMATO SOUP.

One pint of canned tomatoes or four large raw ones, cut up fine. Add one quart of boiling water, and let them boil ten minutes, or until done. Remove from the stove, and stir in one teaspoonful of soda. While foaming, add one pint of sweet milk, salt and pepper, and a small piece of butter. To be eaten hot with crackers, like oyster soup, to which it is almost equal. M. G. R.

---

A BRIGHT little boy, who had been engaged in combat with another boy, was reproved by his aunt, who told him he ought always to wait until the other boy "pitched upon him." "Well," exclaimed the little hero, "but if I wait for the other boy to begin, I'm afraid there won't be any fight."

AGED 128 YEARS.—On the Little Colorado is a lady who avers that she is 128 years of age. She says she was thirty years of age at the time of the dark day which created such consternation. The Spaniards buried all their saints, of which they had a goodly number, while the Indians took to feasting on dogs and other animals. The "dark day" was so called on account of the remarkable darkness which extended throughout America. The obscuration commenced about ten o'clock in the morning of May 19, 1780, and continued until the middle of the next night. Birds sang their evening song, disappeared and remained silent; fowls went to roost, cattle sought the barn-yards, and candles were lighted in the house.

